# Supplementary material for: The longitudinal dynamics and natural history of clonal haematopoiesis
Source: Nature. 2022 Jun 1;606(7913):335–42. doi: 10.1038/s41586-022-04785-z (PMC9177423; doi:10.1038/s41586-022-04785-z)
Supplement: Supplementary file 1 — This file contains Supplementary Methods, notes, figures and references. [file 41586_2022_4785_MOESM1_ESM.pdf]

nature portfolio

https://doi.org/10.1038/s41586-022-04785-z

Supplementary information

The longitudinal dynamics and natural history of clonal haematopoiesis

In the format provided by the authors and unedited

Supplementary Methods

Assessing the predictive performance of clonal growth predictions

Using an additional time-point (phase 6) available for 11 individuals with mutations in *CBL* (c.2434+1G>A), *DNMT3A* (P385fs, R882H, W330X), *GNB1* (K57E), *JAK2* (V617F), *PPM1D* (Q524X), *SF3B1* (K666N, K700E, R625L), *SRSF2* (P95H, P95L), *TET2* (Q1542X) and *U2AF1* (Q157P, Q157R). Using the model described in the "Hierarchical modelling of clone trajectories through time" section of the Methods and conditioning on the previous timepoints, we predict the additional time-point and assess the predictive performance through the mean absolute error (MAE) to the true VAF value.

Validating the dynamic coefficient and age at onset inference with Wright-Fisher simulations

We use Wright-Fisher simulations [1–3] with a fixed population of 200,000 cells and 50 possible drivers, a range of fitness advantages $(0.001 - 0.030)$ and a range of mutation rates $(1.0 * 10^{-10} - 4.0 * 10^{-9})$. These ranges were estimated to cover the values inferred and mentioned in considering that one should expect there to be approximately 13 generations of HSC per year and a population size of 200,000 HSC [4].

To simulate the conditions under which the experimental data was obtained, we fit Gamma distributions to the observed coverage and observed age at first time-point truncated at the minimum and maximum values for each. For each simulation we sample from these distributions the first timepoint, a random number of subsequent timepoints (between 2 and 4) from a uniform distribution and the coverage for each driver at each timepoint. We simulate the sequencing process as drawing samples from a beta-binomial distribution parameterized similarly to the one described in the "Hierarchical modelling of clone trajectories through time" section of the Methods, where the probability is the proportion of cells from a specific clone present at a given time-point. More concretely, $counts \sim BB(\frac{p\beta}{1-p}, \beta, cov)$, where p is the allele frequency of a mutation, $\beta$is the technical overdispersion parameter and cov is the coverage which is sampled from the coverage distribution as inferred from our data.

To infer coefficients under this setting we converted generations to years (13 generations per year) and used the framework described in the previous sections to infer these coefficients. Since the nature of these mutations does not consider different levels of genetic resolution, we had to modify the driver coefficient to $driver\ effect \sim N(0, \sqrt{2 * 0.1^2})$so that the distribution from which this coefficient is being drawn has the one we consider for the driver effect considering a gene, domain and site effect. The observed coefficients are converted to year as $coefficients = (1 + fitness)^g - 1$, where g is the number of generations per year, and we assess the fit between inferred and observed coefficients considering these values. We additionally calculate the age at clone foundation for the inferred coefficients and, using these simulations which allow us to know the true age at clone foundation, we assess the fit between inferred and observed ages at clone foundation.

To better understand the impact that population size and generation times have on these simulations, we conduct the same analysis considering two additional scenarios: a population size of 100,000 HSC and 5 generations per year, and a population size of 50,000 HSC and 1 generation per year.

Finally, we also calculate the age at onset as specified in the "Determining the expected age at beginning of clone onset". To do this, we assume that these clones follow a Wright-Fisher process, where growth can be separated into two distinct phases which depend on the size of the clone - a stochastic phase, where the clone is too small and during which growth happens linearly, and a deterministic phase, during which growth is approximately exponential (**Extended Data Fig. 4a**). According to this growth regime, the age at onset can be calculated as $t_0 adjusted = t_0 + \frac{log(g/b_{total})}{b_{total}} - \frac{1}{b_{total}}$, where t_0 is the age at onset if the clone grew exponentially (as opposed to following a Wright-Fisher process), $\frac{log(g/b_{total})}{b_{total}}$ is the time at which the clone started to grow deterministically and $\frac{1}{b_{total}}$ is the expected time the clone spends following a stochastic growth regime. We assess the validity of this approach by calculating the coefficient of correlation between inferred and true ages at onset from the simulations.

Validating the Phylogenies

We used two established approaches to assess the internal consistency, stability and robustness of the shared mutation data and inferred phylogenetic trees, as used previously [5,6]:

(1) *Assessment of the internal consistency of shared mutation data using the disagreement score.*

A perfect phylogeny contains pairs of mutations that are either in discrete clades or nested one within the other. To test the consistency of our data with this assumption, we calculated a 'disagreement score'. For every pair of loci, we calculated the number of samples in disagreement with this assumption, and then calculated the mean score across all pairs[7]. Samples with unknown genotypes were assumed to be in agreement. Scores from observed phylogenies were then compared to scores generated from random shuffles of the corresponding genotype matrix, internal to each locus. In this way, the number of mutations for each locus is preserved, and the disagreement score in the observed genotype matrix can be compared to the score in the randomly generated matrix. For each of our three phylogenies, the disagreement scores were extremely low at <0.02 in all cases, which was ~100,000-fold lower compared to random shuffles of the genotypes at each locus (**Extended Data Fig. 5d**). This demonstrates that our data have high internal consistency and that the phylogenies are close to what would be expected in a perfect phylogeny, where the disagreement score is 0.

(2) *Comparison of the MPBoot phylogeny with an alternative phylogeny-inference algorithm (SCITE).*

To assess the reliability and stability of the phylogeny inferred by MPBoot, we fed the same genotype data into an alternative algorithm, SCITE[8]. SCITE is a tree-inference algorithm designed for somatic single-cell data that uses Markov chain Monte Carlo sampling with an error model that takes potential false positives and false negatives into account for tree-scoring. We used false positive and false negative rates of 0.001. SCITE produced phylogenies with high agreement to the original MPBoot phylogenies, with Robinson-Foulds similarities of 0.989 (PD34493), 0.996 (PD41305) and 1.000 (PD41276) (**Extended Data Fig. 5e**).

Validating annual growth rate inferences from single-cell phylogenies with Wright-Fisher simulations

We use Wright-Fisher simulations [2,3] with 50 possible drivers and test a range of different fitness advantages ([0.005,0.010,0.015,0.020,0.025,0.030]) over 800 generations at a fixed population size of 200,000 HSC. For each fitness effect we define a driver mutation rate ([$200 * 10^{-9}$,$50 * 10^{-9}$,$20 * 10^{-9}$,$15 * 10^{-9}$,$8 * 10^{-9}$,$5 * 10^{-9}$], respectively) that guarantees that at least a few simulations lead to clones which expand to sufficient sizes and avoid many competing expansions and keep the passenger mutation rate constant ($2 * 10^{-5}$). For each simulation we infer phylogenetic trees by sampling 100 representative clones from our population and using a neighbour-joining algorithm based on mutation presence. The representative sampling is done by defining for each clone a probability of being sampled that is equivalent to its proportion in the population. We then detect the clades that contain drivers, isolate them and infer their effective population size (Neff) trajectory using BNPR [9,10].

We fit different models to the inferred Neff trajectories, namely:

1. A log-linear fit (assumes exponential growth);
2. A scaled and shifted sigmoidal fit (assumes that growth saturates based on the Neff trajectory);
3. A shifted sigmoidal fit (assumes that growth saturates at 1 and that the most recent Neff estimate corresponds to the proportion of tips in the clade);
4. A biphasic log-linear fit (assumes that growth is exponential and has two distinct coefficients corresponding to early and late growth; the boundary between early and late growth - otherwise referred to as the changepoint between both - is also fitted with the other parameters and is constrained to lie in the central part of the trajectory: for the time t over which the clone expands, the changepoint cannot be inferior to $min(t) + 0.25 * range(t)$ nor superior to $max(t) - 0.25 * range(t)$, where $range(t) = max(t) - min(t)$. This constraint prevents fits that are too close to the clonal inception or to the clone at later stages).

We compare these models by assessing how closely they are able to recapitulate the original fitness in the simulations. To do so, we calculate their coefficient of determination and root mean squared error. We also visually assess how similar these trajectories are to the true driver trajectories as reconstructed from simulations - to match clones from a Wright-Fisher simulation to an expansion in a phylogenetic tree we assign each clone from the Wright-Fisher simulation to its nearest clone in a phylogenetic tree using the Hamming distance between the mutations in each clone.

We additionally estimate the effective population size using two other methods for validation - mcmc.popsize and skyline from the ape package [11] in R . This allows us to confirm our observations that stem from phylodynamic estimations and that concern, mostly, a prevalent effect of clonal deceleration which is detailed in the main text and in the following section.

Detecting deceleration in single-cell phylogenies and longitudinal data

We infer the presence of deceleration in both single-cell phylogenies and longitudinal data. To do this, we use two distinct methods: calculating the ratio between the expected and observed VAF and calculating deceleration using growth rates.

For the first method - calculating the ratio between expected and observed VAF - we use the value for the early growth from the changepoint log-linear fit described in “Validating annual growth rate inferences from single-cell phylogenies with Wright-Fisher simulations” and extrapolate the Neff to the age at sampling. By doing so we get the expected clone fraction if growth had not changed during the Neff trajectory. We also calculated the observed clone fraction as the fraction of tips in the clade. To get the expected clone fraction from Neff we divide Neff by the inferred population size in Lee-Six et. al (200,000 HSC) [4]. We then calculate the ratio between the expected and observed clone size - if this ratio is close to 1 this implies little to no changes in dynamics, whereas a ratio above 1 implies deceleration and a ratio below 1 implies acceleration.

For the second method - calculating deceleration using growth rates - we define two distinct quantities for both single-cell phylogenies/longitudinal data - expected/observed growth, corresponding to the growth rate of each clone during observation at old age, and early/minimal historical growth, corresponding to the growth rate of each clone at an earlier stage of clonal dynamics - and calculate the ratio between them.

As such, for phylogenies we first calculate the Neff trajectory for each clade using BNPR [9]. Next, and using their Neff trajectory, we calculate their expected growth rate by assuming a sigmoidal growth. We additionally assume that the final Neff (Neff at sampling) estimate corresponds to the fraction of tips in the clade and we scale our data accordingly such that 1 corresponds to the maximum Neff and the fraction of tips in the clade corresponds to Neff at sampling. Thirdly and using the changepoint log-linear fit described in “Validating annual growth rate inferences from single-cell phylogenies with Wright-Fisher simulations” we derive the value for early growth. Finally, as a measure of deceleration, we calculate the ratio between expected and early growth - a value close to 1 for this ratio implies an absence of deceleration whereas smaller values imply deceleration.

For the longitudinal data we use the observed growth for each clone as described in “Hierarchical modelling of clone trajectories through time”. Next, we calculate the (minimal) historical growth as the growth that excludes all posterior samples that would lead to age at onset estimates exceeding lifetime (ages at onset for clones below -1, a heuristic value chosen to represent developmental onset of clones). Finally and as a measure of deceleration, we calculate the ratio between observed and historical growth. The interpretation for this ratio is similar to that defined in the previous paragraph for phylogenetic data - a value of 1 implies an absence of detectable deceleration, whereas smaller values represent the minimal amount of deceleration. This method has, however a caveat - due to the nature of this calculation (excluding posterior samples which are too slow to provide solutions within lifetime), values above 1 (indicating acceleration) are technically impossible.

Supplementary Notes

Supplementary Note 1 - Determining the effect of repeated sampling on the theoretical limit of detection

Across this work we sequence individuals a median of 4 times across their lifetime. We define a detection threshold of 0.5% VAF as the minimum clone size for detection on individual timepoints, but the repeated sampling leads to 0.5% VAF being an overestimation of the actual limit of detection (LOD) - the size at which clones become detectable.

To show this, we simulate the repeated sampling of variants existing at a true clone proportion between 0 and 2%. We use this proportion p as the probability parameter in a beta binomial distribution, the overdispersion β calculated using technical replicates as the overdispersion in the same beta binomial distribution and a coverage of 1000. Having fully parameterized this distribution ($counts \sim BB(\frac{p\beta}{1-p}, \beta, 1000)$) we sample counts from it between 1 to 5 times. For each combination of clone size and number of samples we perform 1,000 realisations and calculate the number of detected clones at a threshold of 0.5%. This allows us to assess the fraction of clones with a specific size which are detected if we sample them multiple times - in other words, are able to assess the detection rate for different clone sizes and different numbers of samples.

With this, we show that, at a threshold of 0.5% and sampling only once, we detect 14.8% of all clones existing at 0.5% (**Fig. S1**). However, repeating this sampling 3 and 5 times leads to the detection of approximately 37.7% and 54.3% of all clones existing at 0.5%, respectively. As such, under regular conditions - a single sample - we would detect 13.5% of all clones present at 0.5% with a detection threshold of 0.5%. The question we should now ask is: what is the smallest possible clone size we detect at the same rate of detection - 13.5% - if we increase the number of samples? Using the same set of simulations, we can calculate the likely minimal size of the detected clones with clones as small as 0.21% and 0.14% being detected with 3 and 5 samples, respectively, using the same detection rate (with 2 and 4 samples, the theoretical LOD is 0.30% and 0.16%, respectively). As such, when considering the theoretical LOD used in **Fig. 4k**, we avoided using 0.5% which, as we show, would be at least twice as high as the theoretical LOD obtained from simulations.

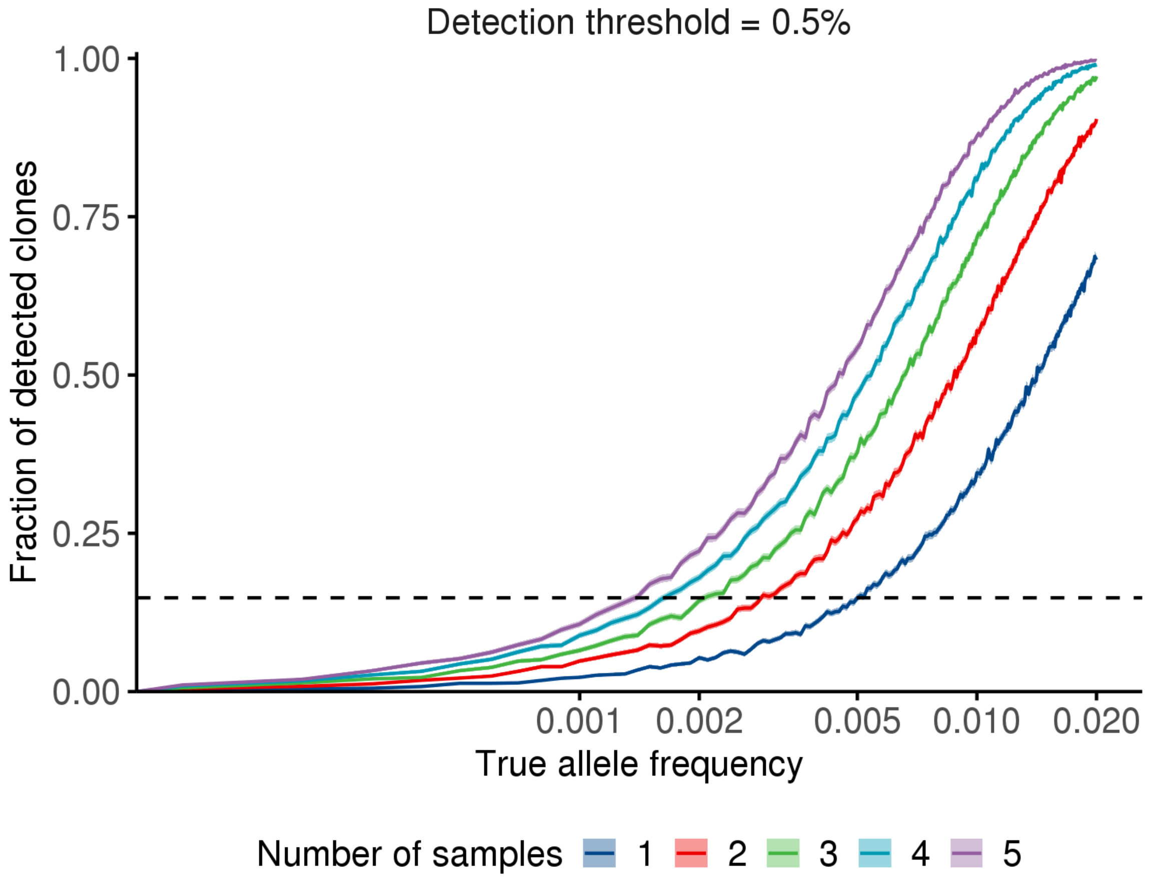

Fig. S1 - Fraction of detected clones upon repeated samples/timepoints at a detection threshold of 0.5%.

Supplementary Note 2 - Patterns of selection in longitudinal and phylogenetic data

In this Supplementary Note we examine changes in selection, as quantified by the dN/dS ratio, in two settings: (i) in our longitudinal cohort, we ask whether dN/dS ratios change with age, and (ii) in our phylogenies, we compare dN/dS ratios in shared and private branches.

We use the dNdScv algorithm, an implementation of dN/dS that corrects for trinucleotide mutation rates, sequence composition, and variable mutation rates across genes [12].

dN/dS ratios in longitudinal data

First, using the 385 individuals included in our longitudinal data set, we compared the dN/dS ratio at the time of study entry (median age 69.3 years) with the ratio at the end-of-study (median age 81.3 years). We derived both global dN/dS ratios across all targeted genes (**Fig. S2**), and gene-specific dN/dS ratios (**Fig. S3**).

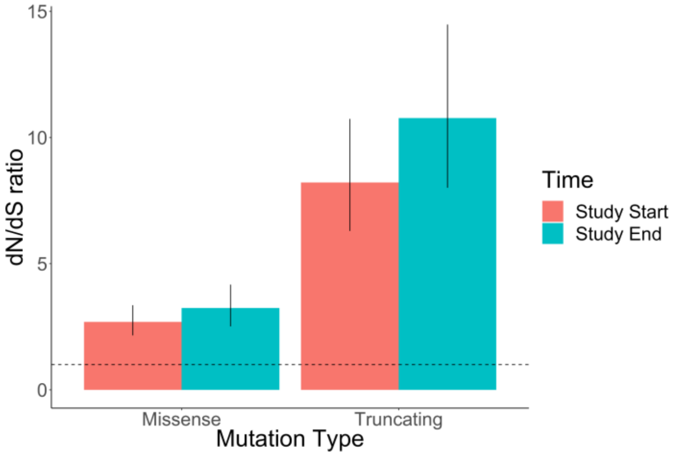

Fig. S2 - Global dN/dS ratios at the start vs end of study. Error bars depict 95% CIs.

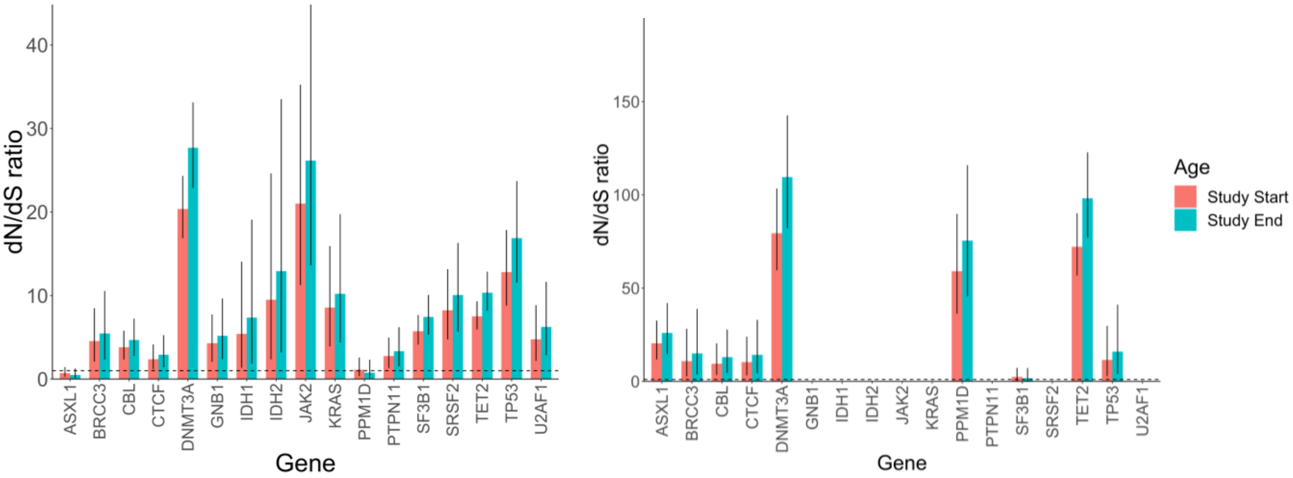

Fig. S3 - Gene-specific dN/dS ratios at the start vs end of study, for missense (left) and truncating (right) mutations. Error bars depict 95% CIs.

We noted a consistent trend for higher dN/dS ratios at older ages. One interpretation of this is that selection tends to strengthen with age. However, we now know from our longitudinal and phylogenetic data that this is not universally true, and that the relationship between age and mutation 'fitness' is gene-specific. For example, we found that *DNMT3A*-mutant clones preferentially expanded early in life and displayed slower growth in old age (suggesting falling selective pressure with advancing age), while splicing gene mutations only drove expansion later in life (suggesting increasing selective pressure with advancing age). Despite this, all driver genes display higher dN/dS ratios at older ages (**Fig. S3**), including, for example, *DNMT3A*. This highlights the inability of dN/dS to quantify selection strength at a particular point in time. Instead, dN/dS reflects the cumulative effects of selection up to the time of sampling. As such, while selection might strengthen with advancing age for many mutations, higher dN/dS ratios at later time-points also reflect the fact that additional mutant clones are identified at older ages, having had longer to reach detectable levels. In this case, drivers are not necessarily 'fitter' in older people, they have simply had longer to expand their cognate clones to a detectable level (by conventional sequencing). This illustrates that dN/dS ratios cannot disentangle the effects of driver fitness and duration of clonal expansion. In contrast, our longitudinal

modelling of clonal trajectories, using serial and phylogenetic data, allows for quantitation of driver mutation fitness specifically.

dN/dS ratios in phylogenetic data

Next we explored how dN/dS ratios vary within haematopoietic phylogenies. Specifically, we asked whether selection strength differs between phylogenetic branches that precede clonal expansions (shared branches, coloured red in **Fig. S4**), as compared to selection along branches that do not (private branches, coloured grey in **Fig. S4**).

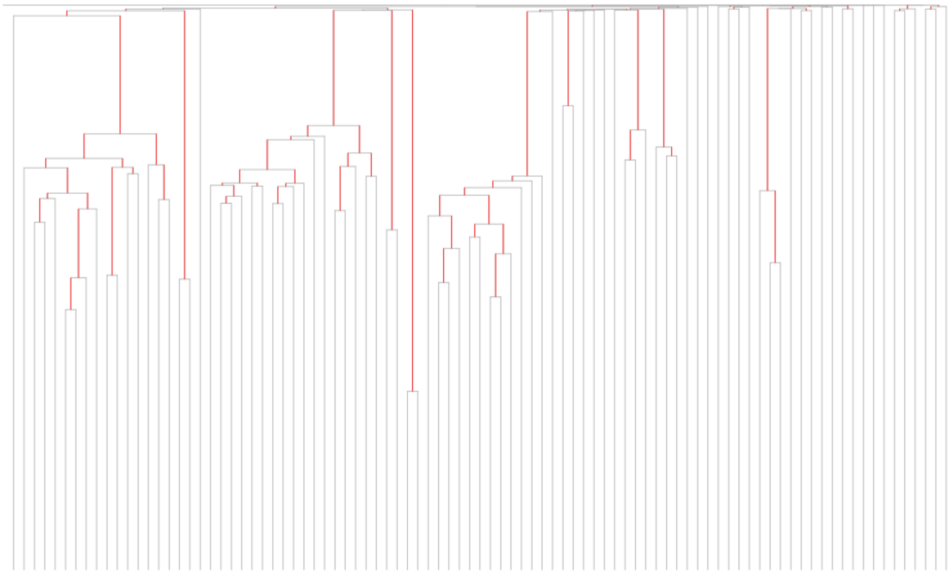

Fig. S4. Example phylogeny (PD41305) illustrating the distinction between shared (red) and private (grey) branches.

We combined our three phylogenies (as depicted in **Fig. 3** of the main manuscript) into a single analysis to maximise our power to detect signal from the limited number of coding mutations across the genomes of just 3 individuals (total of 245 shared and 1885 private somatic mutations). Applying the dNdScv algorithm, we observe a trend towards higher global dN/dS ratios along shared vs private branches (**Fig. S5**).

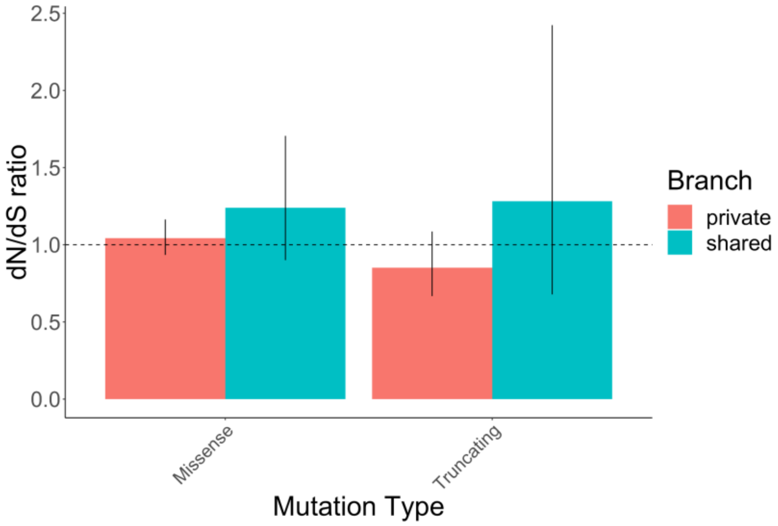

Fig. S5. Global dN/dS ratios among shared vs private mutations. Error bars depict 95% CIs.

Since mutations acquired along shared phylogeny branches are, by definition, those preceding clonal expansion, it is expected that selection would be strongest here, as compared to along private branches, where mutations do not, by definition, lead to clonal expansion. Excluding known CH driver mutations (defined as those in the 17 genes included in our longitudinal model), or excluding any mutation in a cancer gene (defined as those in Tier 1 of the Cancer Gene Census; https://cancer.sanger.ac.uk/census), made almost no impact on the dN/dS ratios derived as above. This is consistent with the existence of clones without known drivers in elderly individuals, and our observation that such clones grow over their lifetimes at rates comparable to clones with known drivers (**Fig. 3**, main manuscript).

Supplementary Note 3 - Investigating clonality and its possible impact on inference

In this Supplementary Note, we tentatively infer co-clonality (whether two or more mutations are in the same clone) and investigate the potential impact this may have on our inference of clonal growth.

Clonality inference from targeted sequencing data

Our initial assumption, backed up by single-cell studies[13], was that CH clones commonly harbour a single driver mutation. Nonetheless, we try here to investigate the possibility that two or more driver mutations occur in the same clone. Typically, clonality can be successfully inferred when information on large parts of the genome is available (e.g. with whole genome or exome sequencing). The longitudinal part of our study required deep sequencing of 56 genes in 1593 samples, such that we needed to focus our sequencing on the relevant genes in order to robustly track small and large clones over time. Unfortunately, such highly targeted sequencing does not normally provide sufficient information to facilitate clonality inferences. Nevertheless, here we attempt a number of heuristic tests to investigate this specifically using the following tools/approaches:

- **PyClone** - PyClone is a software tool that relies on copy number data and deep sequencing to infer the clonal architecture of a sample [14];
- **Fisher test consistency** - if mutation counts are consistently identical (i.e. non-significant in a Fisher's test) through time, it becomes more likely that they are present in the same clone;
- **Pigeonhole principle** - if the sum of VAF for a set of heterozygous mutations is greater than 50%, it is biologically impossible for them to be all in separate clones;
- **Dynamic similarity** - if two mutations are dynamically similar (i.e. their annual growth rates are similar) this suggests that they may reside in the same clone.

On clonal structure

Here, it should be noted that, in the absence of genome-scale data, it is possible that clustering mutations by relative frequencies can be indicative of the following:

1. Two separate but similarly sized clones (false positive);
2. A clone carrying one mutation acquires a second mutation (true positive);
3. A parental clone with one or more unknown drivers acquires one or more known drivers in the same subclone (true positive);
4. A parental clone with one or more unknown drivers acquires two known drivers in distinct subclones: here, while both new drivers are acquired in cells of the ancestral clone (marked by the unknown driver), each are actually in distinct subclones (false positive).

For cases 3 and 4 (represented in Fig. S6), we should be clear in our nomenclature as both imply that "two mutations are in the same clone" but only one of them is of relevance (3). As such and for clarity,

we are interested in detecting 3 and this is what we mean by "two mutations in the same clone"; 4 should be further specified and defined as "two mutations in separate subclones in the same parental clone".

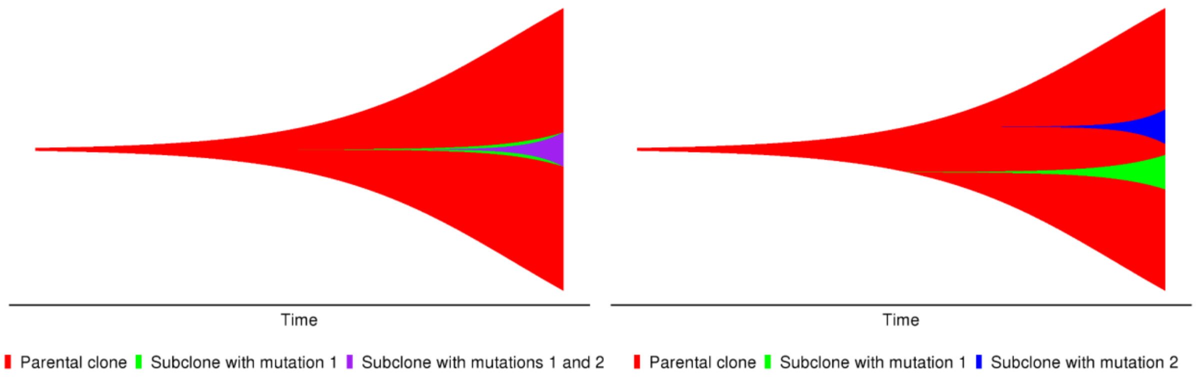

Fig. S6 - Representation of two different scenarios of possible confusion when referring to “two mutations being in the same clone”. On the left, two consecutive mutations are acquired in the same parental clone and subclone, while on the right two mutations are acquired in separate subclones, having nonetheless similar sizes.

PyClone analysis

PyClone[14] typically relies on deeply sequenced somatic mutations and copy number information to phase mutations into specific clones. However, our data is limited for this purpose, given the very small number of somatic mutations identified in each individual’s blood DNA (median 2). Indeed, if we analyse our longitudinal data using PyClone (the PyClone input for each individual were their respective mutation calls for all timepoints), the limited input data leads the software to propose co-clonality in *all* individuals with more than one mutation. Furthermore, for the 76 individuals with 3 mutations, Pyclone proposed that all 3 belonged to just one clone in 67% of cases (51/76). These inferences are implausible and almost certainly a consequence of the limited input mutation data. In fact, evidence suggests that CH mutations are not commonly co-clonal, including the following. *First*, a recent study used single-cell DNA sequencing to specifically determine whether CH driver mutations existed in separate cells or coexisted in the same cell [13]. In this study, individuals with CH never harboured more than one mutation in epigenetic regulator genes in the same cell/clone (including in the two most common CH driver genes, *DNMT3A*, *TET2*, and also *ASXL1* and *IDH1/2*). Also, only 2/21 (9.5%) individuals were found to harbour more than one CH mutation in the same cell/clone. *Second*, upon close inspection, several instances of co-clonality proposed by PyClone in our data are functionally implausible. For example, there were instances of putative co-clonal mutations that are known to not co-occur in the same cell (e.g. *SF3B1* and *U2AF1* mutations, proposed by PyClone to exist in the same clone in one individual, but previously shown to be mutually exclusive[15]). For these reasons, we did not use Pyclone for clonality inference as it is not a suitable tool for our data.

Longitudinal consistency of Fisher's test

We used a Fisher's test to compare the VAFs of any two mutations within an individual. If this comparison was consistently non-significant over time (i.e. the VAFs of the two mutations did not differ), we considered these two mutations to be putatively in the same clone. For each time-point and for each mutation pair within an individual, we tested for significance according to a Fisher's test

using a contingency table with the number of mutant and reference reads for each mutation. We define potential co-clonality (i.e. two mutations in the same clone) in cases where the Fisher's test is not significant at all time-points. Using this definition, out of 752 mutation pairs, 102 are putatively co-clonal.

Importantly, while this analysis detects clones which are consistently similar in size over a median period of 13 years (consistent with co-clonality), it does not exclude the alternative (and realistic) scenario that two independent clones show similar growth trajectories within an individual.

Pigeonhole principle

The pigeonhole principle states that if the combined VAFs of a group of mutations within an individual is greater than the carrying capacity of the population then the mutations cannot be in distinct clones. Applying this principle, we found only six pairs of mutations that are proposed to exist in the same clone.

Dynamic similarity

If two mutations have similar annual growth rates, they may be in the same clone. To assess this, we subtract the posterior samples for growth rate of every mutation in a mutation pair *within* an individual. If the difference in coefficients is close to 0, this makes co-clonality of the two mutations more likely. To obtain a background distribution, we compare random mutation pairs *between* individuals (i.e. the growth rates from two distinct mutations in different individuals). As shown in Fig. S7, the distribution of differences in growth coefficients between any two randomly selected mutations is practically identical to that of the difference in growth coefficients between two mutations within the same individual. Therefore, this approach is not adequate for determining clonality.

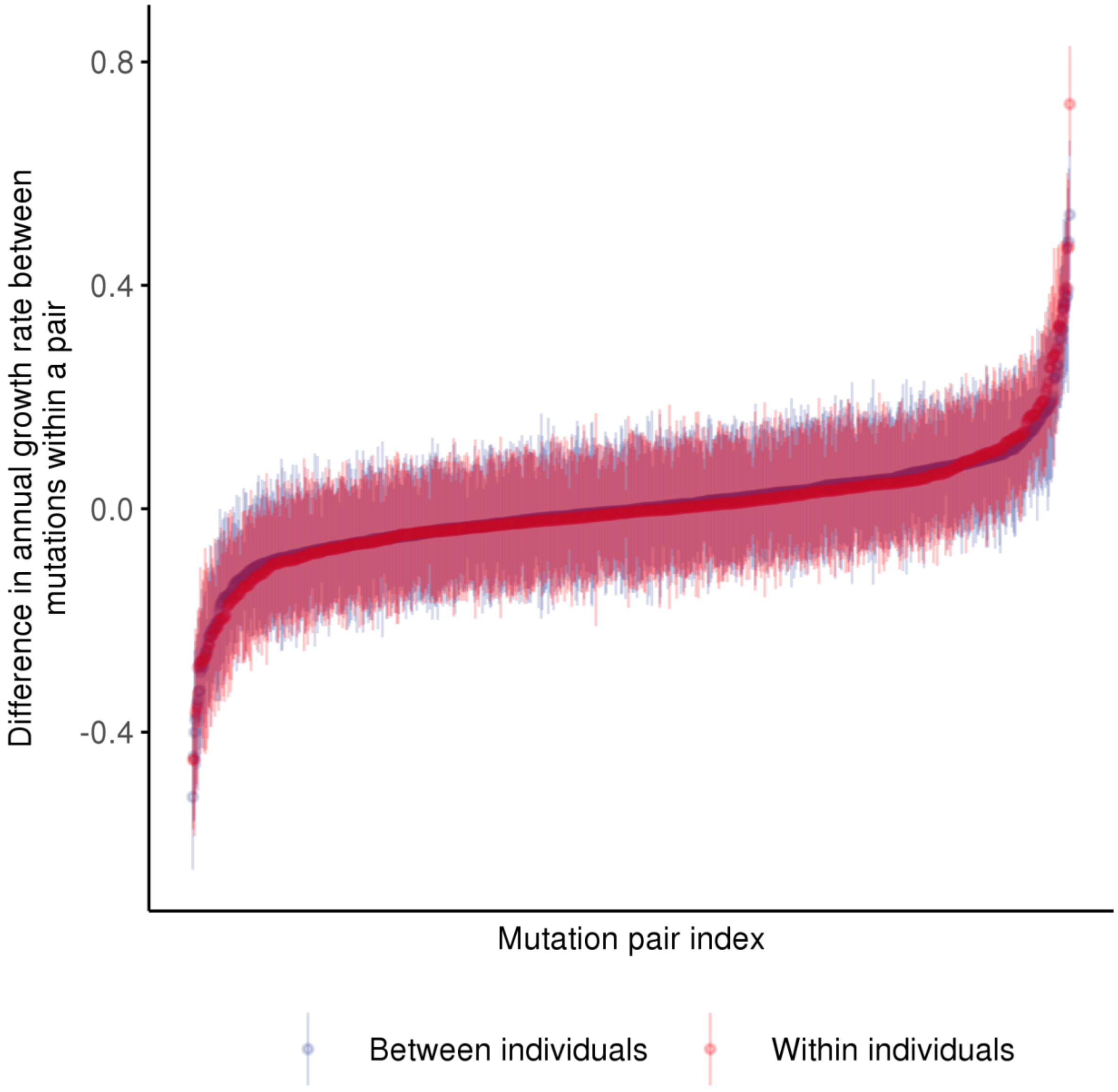

Fig. S7 - Distribution of differences in growth rates between mutations within pairs, where the two mutations are either (i) present in the same individual (*within* individuals) or (ii) randomly selected from our cohort (*between* individuals). The point estimates represent the average value of the difference, whereas the intervals represent the 90% confidence interval for the difference.

The impact of clonality on the inference of clonal growth rates

As discussed above, our data do not allow us to determine co-clonal events with any confidence. Importantly, this lack of certainty would be of no tangible consequence for our modeling of clonal growth rates, if potential co-clonality had no impact on our inferences of CH growth dynamics. To assess this, we checked whether there was any association between the number of clones within an individual (where multiple mutations could be consistent with co-clonal events) and: **(i)** the unknown-cause growth (i.e. the component of overall clonal growth not accounted for by the identity of the driver mutation) and **(ii)** the fraction of clonal trajectories growing at a constant rate over time (referred to here as 'explained trajectories'). As observable in Fig. S8, no such associations were detectable.

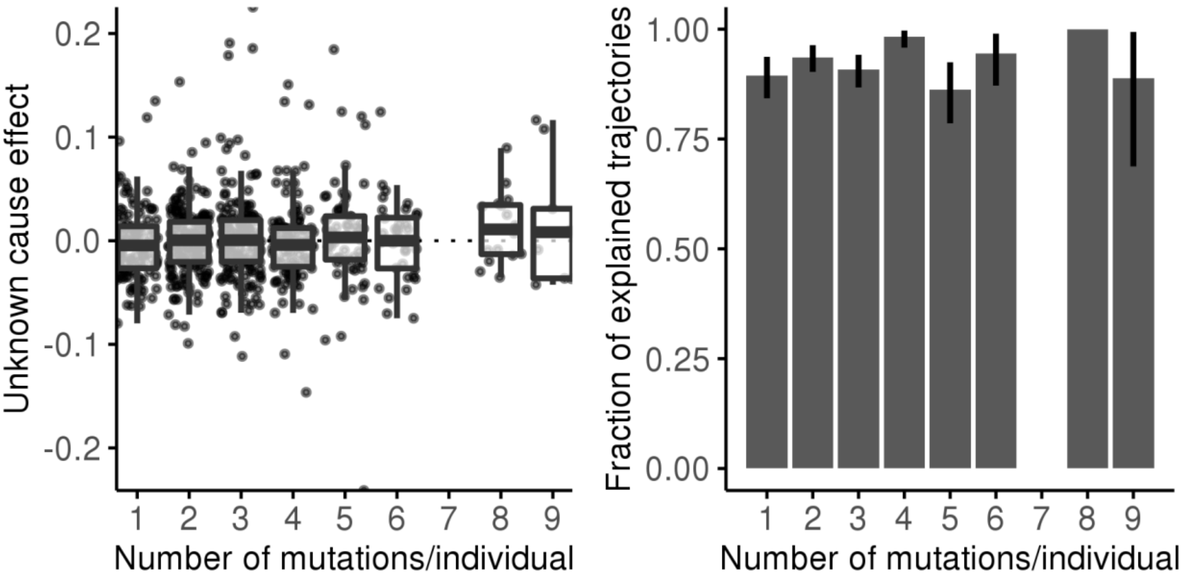

Fig. S8 - Association between number of mutations per individual and unknown cause effect (left) and fraction of explained trajectories (right; n=685). The boxes represent the 25th, 50th (median) and 75th percentiles of the data; the whiskers represent the lowest (or highest) datum within 1 interquartile range from the 25th (or 75th) percentile. Intervals in the right panel are beta-distributed 90% confidence intervals.

We confirmed this lack of a relationship between the number of mutations and growth rate in *DNMT3A* or *TET2* (the two genes for which sufficient numbers are available for this comparison), where we find no association between the number of mutations in a given individual and the average *DNMT3A* or *TET2* growth rate (R=-0.12, p=0.15 and R=0.03, p=0.68, respectively).

In conclusion, while there may be a small number of clones that harbour more than one mutation, these do not affect our inferences relating to CH clonal dynamics [5,13].

Supplementary References

1. Gillespie, J. H. *Population Genetics: A Concise Guide*. (JHU Press, 2004).
2. Beerenwinkel, N. & Gerstung, M. *clonex*. (Github).
3. Beerenwinkel, N. *et al.* Genetic progression and the waiting time to cancer. *PLoS Comput. Biol.* **3**, e225 (2007).
4. Lee-Six, H. *et al.* Population dynamics of normal human blood inferred from somatic mutations. *Nature* **561**, 473–478 (2018).
5. Mitchell, E. *et al.* Clonal dynamics of haematopoiesis across the human lifespan. *bioRxiv* (2021).
6. Spencer Chapman, M. *et al.* Lineage tracing of human development through somatic mutations. *Nature* **595**, 85–90 (2021).
7. Planet, P. J. Tree disagreement: measuring and testing incongruence in phylogenies. *J. Biomed. Inform.* **39**, 86–102 (2006).
8. Jahn, K., Kuipers, J. & Beerenwinkel, N. Tree inference for single-cell data. *Genome Biol.* **17**, 86 (2016).
9. Karcher, M. D., Palacios, J. A., Lan, S. & Minin, V. N. phylodyn: an R package for phylodynamic simulation and inference. *Mol. Ecol. Resour.* **17**, 96–100 (2017).
10. Lan, S., Palacios, J. A., Karcher, M., Minin, V. N. & Shahbaba, B. An efficient Bayesian inference framework for coalescent-based nonparametric phylodynamics. *Bioinformatics* **31**, 3282–3289 (2015).
11. Paradis, E. & Schliep, K. ape 5.0: an environment for modern phylogenetics and evolutionary analyses in R. *Bioinformatics* **35**, 526–528 (2019).
12. Martincorena, I. *et al.* Universal Patterns of Selection in Cancer and Somatic Tissues. *Cell* **171**, 1029–1041.e21 (2017).
13. Miles, L. A. *et al.* Single-cell mutation analysis of clonal evolution in myeloid malignancies. *Nature* **587**, 477–482 (2020).

14. Roth, A. *et al.* PyClone: statistical inference of clonal population structure in cancer. *Nat. Methods* **11**, 396–398 (2014).

15. Thol, F. *et al.* Frequency and prognostic impact of mutations in SRSF2, U2AF1, and ZRSR2 in patients with myelodysplastic syndromes. *Blood* **119**, 3578–3584 (2012).
