# Supplementary material for: The longitudinal dynamics and natural history of clonal haematopoiesis
Source: Nature. 2022 Jun 1;606(7913):335–42. doi: 10.1038/s41586-022-04785-z (PMC9177423; doi:10.1038/s41586-022-04785-z)
Supplement: Supplementary file 2 — Reporting Summary [file 41586_2022_4785_MOESM2_ESM.pdf]

nature portfolio

Corresponding author(s): George S. Vassiliou & Moritz Gerstung

Last updated by author(s): Feb 2, 2022

Reporting Summary

Nature Portfolio wishes to improve the reproducibility of the work that we publish. This form provides structure for consistency and transparency in reporting. For further information on Nature Portfolio policies, see our Editorial Policies and the Editorial Policy Checklist.

Statistics

For all statistical analyses, confirm that the following items are present in the figure legend, table legend, main text, or Methods section.

n/a	Confirmed	
☐	☒	The exact sample size (*n*) for each experimental group/condition, given as a discrete number and unit of measurement
☐	☒	A statement on whether measurements were taken from distinct samples or whether the same sample was measured repeatedly
☐	☒	The statistical test(s) used AND whether they are one- or two-sided<br>*Only common tests should be described solely by name; describe more complex techniques in the Methods section.*
☐	☒	A description of all covariates tested
☐	☒	A description of any assumptions or corrections, such as tests of normality and adjustment for multiple comparisons
☐	☒	A full description of the statistical parameters including central tendency (e.g. means) or other basic estimates (e.g. regression coefficient) AND variation (e.g. standard deviation) or associated estimates of uncertainty (e.g. confidence intervals)
☐	☒	For null hypothesis testing, the test statistic (e.g. *F*, *t*, *r*) with confidence intervals, effect sizes, degrees of freedom and *P* value noted<br>*Give P values as exact values whenever suitable.*
☐	☒	For Bayesian analysis, information on the choice of priors and Markov chain Monte Carlo settings
☐	☒	For hierarchical and complex designs, identification of the appropriate level for tests and full reporting of outcomes
☐	☒	Estimates of effect sizes (e.g. Cohen's *d*, Pearson's *r*), indicating how they were calculated

Our web collection on statistics for biologists contains articles on many of the points above.

Software and code

Policy information about availability of computer code

Data collection	Image processing from sequencing data using standard Illumina NovaSeq and HiSeq pipelines.
Data analysis	Read alignment was performed using BWA-MEM (v0.7.17). Somatic mutation calling and VAF quantification was performed using Shearwater (v.1.21.5), CaVEMan (v.1.11.2), Pindel (v.2.2) and the in-house script vafCorrect (https://github.com/cancerit/vafCorrect). Allele counts at recurrent mutation hotspots were verified using an in-house script (github.com/cancerit/allelecount). Copy number aberrations in blood colonies WGS were determined using ASCAT (v4.2.1) and BRASS (https://github.com/cancerit/BRASS). Phylogenetic trees were derived using MPBoot (version 1.1.0 for Linux (http://www.iqtree.org/mpboot)) and treemut (https://github.com/NickWilliamsSanger/treemut) and validated using SCITE (https://gitlab.com/jahnka/SCITE). Population size trajectories were determined using Bayesian nonparametric phylodynamic reconstructions (BNPR) implemented in the phylodyn (v0.9.2) package for R. dNdScv (v.0.0.1.0) was used to quantify selection for somatic mutations. Wright-Fisher simulations of the haematopoietic system were performed using clonex (https://github.com/gerstung-lab/clonex).<br>All statistical analyses used the software R (v.3.6.3). All R files used for the longitudinal and phylodynamic modelling and validation are publicly available at https://github.com/josegcpa/clonal_dynamics. All files used for the construction of phylogenetic trees are publicly available at https://github.com/margaretefabre/Clonal_dynamics.<br>A list of all the R packages explicitly used (excluding package prerequisites) and their respective versions (in brackets) is provided here: MASS (v7.3), ape (v5.4), bayesplot (v1.7.2), car (v3.0), castor (v1.6.4), coda (v0.19), colorspace (v1.4), cowplot (v1.1.0), dendextend (v1.14.0), extraDistr (v1.9.1), ggforce (v0.3.2), ggpubr (v0.4.0), ggrepel (v0.8.2), ggridges (v0.5.2), ggsci (v2.9), ggsignif (v0.6.0), ggtree (v2.0.4), greta (v0.3.1), gridExtra (v2.3), gtools (v3.8.2), nlme (v3.1), openxlsx (v4.2.2), phangorn (v2.5.5), phylodyn (v0.9.2), reghelper (v1.0.1), rreticulate (v1.16), scatterpie (v0.1.5), stringr (v1.4.0), survival (v3.2), survminer (v0.4.8), tensorflow (v2.2.0), tidyverse (v1.3.0), zoo (v1.8), deepSNV (v1.21.5) and dNdScv (v.0.0.1.0).

For manuscripts utilizing custom algorithms or software that are central to the research but not yet described in published literature, software must be made available to editors and reviewers. We strongly encourage code deposition in a community repository (e.g. GitHub). See the Nature Portfolio guidelines for submitting code & software for further information.

Data

Policy information about availability of data

All manuscripts must include a data availability statement. This statement should provide the following information, where applicable:

- Accession codes, unique identifiers, or web links for publicly available datasets
- A description of any restrictions on data availability
- For clinical datasets or third party data, please ensure that the statement adheres to our policy

The data files necessary to run the analysis in https://github.com/josegcpa/clonal_dynamics are freely available at https://doi.org/10.6084/m9.figshare.15029118. All sequencing data have been deposited in the European Genome-phenome Archive (EGA) (https://www.ebi.ac.uk/ega/). Targeted sequencing data have been deposited with EGA accession numbers EGAD00001007682 and EGAD00001007683; WGS data have been deposited with accession number EGAD00001007684. Data from the EGA are accessible for research use only to all bona fide researchers, as assessed by the Data Access Committee (https://www.ebi.ac.uk/ega/about/access). Data can be accessed by registering for an EGA account and contacting the Data Access Committee.

AML datasets were retrieved from Papaemmanuil et al, NEJM, 2016 (Ref...)
MDS datasets were retrieved from Papaemmanul et al, Blood 2013 (Ref...)
Phylogenetic data from the Mitchell et al companion paper was kindly shared with us from the authors of the paper.

Field-specific reporting

Please select the one below that is the best fit for your research. If you are not sure, read the appropriate sections before making your selection.

☒ Life sciences ☐ Behavioural & social sciences ☐ Ecological, evolutionary & environmental sciences

For a reference copy of the document with all sections, see nature.com/documents/nr-reporting-summary-flat.pdf

Life sciences study design

All studies must disclose on these points even when the disclosure is negative.

Sample size	To enable us to derive robust estimates of clonal dynamics over time we wanted to study a sufficient number of individuals with clonal haematopoiesis driven by each of its 9 most common driver genes (DNMT3A, TET2, ASXL1, SF3B1, TP53, SRSF2, PPM1D, JAK2 and U2AF1). Our expectation was that clones driven by mutations in the same gene would behave relatively similarly, but the extent of similarity was not known at the outset of the study. We therefore aimed to capture at least 6-8 cases of CH driven by each of these genes. As U2AF1 is the least commonly mutated of these genes (approximately 2-3% of individuals in the studied age range), this was the gene that determined our sample size of 385 participants, which gave an expectation that we would identify approximately 7-12 individuals with U2AF1-driven clonal haematopoiesis. In the end we identified 8 such cases, whilst numbers of individuals with mutations in each of the other 8 genes were greater.
Data exclusions	We excluded DNA sequencing reads that did not meet widely accepted quality metrics. We also excluded 16 single cell colony-derived WGS data that did not meet set quality criteria.
Replication	This is the first study to sequence serially obtained samples in order to study the dynamic behaviour of clonal haematopoiesis. As such, no comparable datasets are available for replication.
Randomization	N/A - there was no intervention or treatment studied.
Blinding	N/A - there was no intervention or treatment studied.

Reporting for specific materials, systems and methods

We require information from authors about some types of materials, experimental systems and methods used in many studies. Here, indicate whether each material, system or method listed is relevant to your study. If you are not sure if a list item applies to your research, read the appropriate section before selecting a response.

Materials & experimental systems

n/a	Involved in the study	
☒	☐	Antibodies
☒	☐	Eukaryotic cell lines
☒	☐	Palaeontology and archaeology
☒	☐	Animals and other organisms
☐	☒	Human research participants
☒	☐	Clinical data
☒	☐	Dual use research of concern

Methods

n/a	Involved in the study	
☒	☐	ChIP-seq
☒	☐	Flow cytometry
☒	☐	MRI-based neuroimaging

Human research participants

Policy information about studies involving human research participants

Population characteristics	The 385 study participants (199 women) were aged 54-93 years (median 69.3) at study entry and were sampled up to 5 times (median 4) over 3.2-16 years (median 12.9 years). The participants had no history of haematological malignancy, but were otherwise unselected.
Recruitment	Participants were recruited as part of the Sardinia study. The study recruited unselected Sardinians aged 14-102 years with the initial sample cohort of >6000 people including over 62% of the eligible population living in the catchment region in Ogliastra. Recruitment of the individuals in the cohort occurred prior to the large studies by us and others reporting the frequency of clonal haematopoiesis and the commonly mutated driver genes in 2014-2015. Our study investigated 385 individuals aged 54 years or older, who were randomly selected from Sardinia study participants without any identifiable selection bias (see - https://sardinia.nia.nih.gov/). Individuals who developed a haematological malignancy before or after recruitment were excluded from the present study.
Ethics oversight	Ethical permission for this study was granted by The East of England (Essex) Research Ethics Committee (REC reference 15/EE/0327). All applicants signed informed consent, which is now stated clearly in the manuscript.

Note that full information on the approval of the study protocol must also be provided in the manuscript.
